# Performance of broilers fed diets supplemented with two yeast cell wall strains using two feeding strategies

**DOI:** 10.1002/vms3.172

**Published:** 2019-04-10

**Authors:** Mohammed M. Hashim, Hector E. Leyva‐Jimenez, Morouj N. Al‐Ajeeli, Yasser J. Jameel, Thomas A. Gaydos, Christopher A. Bailey

**Affiliations:** ^1^ Department of Poultry Science Texas A&M University System College Station Texas USA; ^2^ H.J. Baker & Bro., LLC Shelton Connecticut USA; ^3^ Phileo‐Lesaffre Animal Care Milwaukee Wisconsin USA

**Keywords:** yeast cell wall, broilers, *Saccharomyces cerevisiae*, prebiotic

## Abstract

Different supplements or strategies have been proposed as alternatives to the use of antibiotics at sub‐therapeutic levels in chickens. Mannan oligosaccharides and *β*‐glucans, yeast cell wall fractions (YCW), have been reported to beneficially influence broiler performance and health. Two differently produced yeast cell wall fractions derived from *Saccharomyces cerevisiae* were evaluated in this study using two different supplementation strategies offered to full‐term broilers. The birds were placed in floor pens on used pine‐shaving litter to increase potential microbial stress and mimic industry practice. The study utilized a three‐phase feeding program with a 1‐ to 21‐day starter, 21‐ to 35‐day grower and 35‐ to 42‐day finisher phases. Five dietary treatments were compared in this study. The experimental diets consisted of a control basal broiler diet; or the basal diet supplemented with the two differently produced fractions of YCW. The YCW products were supplemented at a constant 250 ppm or a decreasing concentration program (500, 250, 125 ppm) throughout the three feeding phases. Birds fed diets supplemented with either YCW products at any inclusion regimen demonstrated higher (P < 0.05) body weight (BW) in all three phases than control birds. The difference in final 42‐day BW of the YCW treatments (3041 g) averaged 165 g higher (*P* < 0.05) than the control group. For all YCW treatments, productivity index was higher (*P* < 0.05) in the grower (418) and finisher phase (441) versus control birds (389 grower and 415 finisher). These results suggested that both YCW fractions prepared from *Saccharomyces cerevisiae* can improve broiler performance when added at either a constant rate (250 ppm) or at a decreasing rate from 500 ppm for the starter to 125 ppm for the finisher phase.

## Introduction

In the poultry industry, it is common to reuse old litter for more than one cycle (Coufal *et al*. 2006; Wang *et al*. 2016). However, the old litter provides a suitable environment for different kinds of living organisms, including pathogenic microbes such as coccidia (Lu *et al*. 2003). In addition, increased humidity and pH at warm temperatures enhance the proliferation of pathogenic bacteria in litter (Cressman *et al*. 2010). The use of antibiotics at sub‐therapeutic levels in animal feed as growth promoters improves animal performance and resistance to pathogens (Dibner & Richards 2005). The application of antimicrobials as growth promoters has been employed in the livestock industry for more than 70 years (Dibner & Richards 2005; Sun *et al*. 2005). However, concerns regarding the development of antibiotic‐resistant bacteria in animals and potential transfer to humans have introduced the complete ban of antibiotics as growth promoters in the European Union since 2006, and increased demand for alternatives (Mathur & Singh 2005; Castanon 2007). This is especially important at this time in the U.S. given the Veterinary Feed Directive banning over‐the‐counter use of human important antibiotics in animal feeds went into effect in the year 2017.

Supplementation of yeast cell wall products in animal feed has been reported as a potential alternative to antimicrobial growth promotors (Hashim *et al*. 2013; Fowler *et al*. 2015a). Yeast cell wall is capable of changing the microflora community structure in the gastrointestinal tract and changing the histological structure of the gut mucosa (Reisinger *et al*. 2012; M'Sadeq *et al*. 2015). Antimicrobial peptides, important components of the innate immune response, express antimicrobial and immunomodulatory characteristics and are significantly upregulated in the jejunum due to the glucans of YCW (Tian *et al*. 2016).

Different production methods and sources of YCW, such as brewer's and baker's yeast, have been used in the poultry industry. These product differences are likely one of the reasons for different outcomes when YCW is added to animal diets (Fowler *et al*. 2015b). Studies conducted by our laboratory have demonstrated positive effects of YCW on starter broiler performance under *Clostridium perfringens* (Cp) challenge (Fowler *et al*. 2015b). However, these studies were conducted using battery cages that are not similar to commercial settings and cannot extend beyond 24 days because of cage size restriction. Therefore, the current research focused on studying broiler performance using two differently produced YCW fractions derived from *Saccharomyces cerevisiae* fed in commercial‐like settings using two feeding strategies, constant rate feeding at 250 ppm and decreasing rate feeding at 500, 250 and 125 ppm for starter, grower and finisher phases over a 42‐day rearing period. We hypothesized that YCW prebiotics can improve the performance of broilers using reused litter as an industry‐type pathogen challenge.

## Materials and methods

### Dietary treatments

Two proprietary YCW products extracted from *Saccharomyces cerevisiae* were used in this study to prepare the dietary treatments (Phileo‐Lesaffre Animal Care, Milwaukee, WI). A basal corn/soy‐based broiler diet (Table 1) was formulated during each phase (starter, grower and finisher) to meet the nutrient requirements suggested by the Ross‐308 management handbook (Aviagen, 2014). The diet was then divided into five equally sized batches that included a control (basal diet only, T1); the basal diet plus 250 ppm YCW‐1 at a constant rate during the whole trial (T2); the basal diet plus YCW‐1 at 500 ppm in the starter phase, 250 ppm in grower phase and 125 ppm in finisher phase (T3); the basal diet plus 250 ppm YCW‐2 at a constant rate during the whole trial (T4); and the basal diet plus YCW‐2 at 500 ppm in the starter phase, 250 ppm in the grower phase and 125 in the finisher phase (T5).

**Table 1:** Composition and nutrient content of the experimental basal diets

Ingredient (%)	Starter 1–21 days	Grower 21–35 days	Finisher 35–42 days
Corn	61.6	66.57	70.45
Dehulled soybean meal	31.7	27.20	22.84
Dl‐Methionine 98%	0.23	0.24	0.11
Lysine HCl	0.19	0.16	0.13
Soybean oil	2.20	2.21	3.15
Limestone	1.44	1.48	1.35
Mono‐dicalcium phosphate (%)	1.55	1.39	1.38
Salt	0.51	0.41	0.14
Trace minerals premix^a^	0.05	0.05	0.05
Vitamin premix^b^	0.25	0.25	0.25
Coban‐90 (Monensin)	0.05	0.05	‐
Sodium bicarbonate	‐	‐	0.17
Calculated nutrient content (%)			
Crude Protein	22.00	20.00	18.00
ME (MJ kg^−1^)	12.76	12.97	13.39
Crude fat	3.94	4.04	4.99
Crude fiber	2.14	2.02	1.90
Calcium	0.95	0.92	0.85
Available phosphate	0.45	0.41	0.40
Sodium	0.22	0.18	0.12
Methionine	0.56	0.54	0.39
Lysine	1.31	1.15	1.00

a Trace minerals provided the following per kilogram of diet: Cu, 7.0 mg; I, 0.4 mg; Fe, 60.0 mg; Mn, 60.0 mg; Zn, 60.0 mg.

b Vitamin premix added at this rate yields (kg^−1^): vitamin A, 11 kIU; vitamin D_3_, 3850 IU; vitamin E, 45.8 IU; menadione, 1.5 mg; B_12_, 0.017 mg; biotin, 0.55 mg; thiamine, 2.93 mg; riboflavin, 5.96 mg; d‐pantothenic acid, 20.17 mg; B_6_, 7.15 mg; niacin, 45.8 mg; folic acid, 1.74 mg; choline, 130.3 mg.

### Animal husbandry

The study was conducted at the Poultry Science Research Center and was approved by the Texas A&M Institutional Animal Care and Use Committee (IACUC 2014‐0030). A total of 960 straight‐run Ross‐308 broiler chicks were randomly assigned to 60 3.34 m^2^ floor pens with 16 birds/pen in a completely randomized block design with 12 pen replicates per treatment. The birds were placed in floor pens on used pine‐shaving litter that was used for one previous flock. The study utilized a full‐term 42‐day, three‐phase rearing program with a 21‐day starter, 14‐day grower and 7‐day finisher phases. Mortality within the first 3 days was replaced with new birds that had been fed the control diet. Feed and water were provided *ad libitum* using hanging bucket feeders and nipple drinkers. Mortality was monitored daily.

### Collected data

Average body weight (BW) and feed intake per pen were collected for each feeding phase to evaluate performance. The pen served as the experimental unit for this study. The total birds in each pen were weighed at the beginning and end of each phase to calculate average bird weight per pen. Total feed offered and remaining uneaten feed were calculated at the beginning and end of each phase to calculate the average feed consumption per bird per pen. Studied variables include: BW, body weight; WG, weight gain; F:G, feed to gain ratio; C‐F:G, cumulative feed to gain; FCR, feed to weight; PI, productivity index (livability (%) x BW (kg)/Age (d)/FCRx100); Mort, percent mortality; C‐Mort, cumulative percent mortality.

### Statistical analysis

The experiment was designed as a completely randomized block with five treatments in 12 blocks (pen location within the rearing house). All performance data except mortality were analysed by a one‐way ANOVA using the GLM procedure of SPSS (IBM, Armonk, NY) (SPSS, 2010). Mortality among all treatment groups was compared using chi‐square test (*P* < 0.05). All mortality data were based on the total number of birds dying per treatment group and analysed using SPSS (SPSS, 2010). The total number of birds in each treatment is 192. Therefore, each bird counts for 0.52% mortality. Means deemed significant at *P* ≤ 0.05 were separated using a protected Duncan's Multiple Range Test (Duncan 1955).

## Results

The starter phase results are demonstrated in Table 2 (mean ± standard deviation). At the end of the 21‐day starter phase, birds that were fed diets supplemented with either of the YCW products at any dietary concentration performed better than the control birds. The BW of birds in T2, T3, T4 and T5 was higher (*P* < 0.05) than BW of birds in the control group (819 ± 54, 830 ± 51, 807 ± 38 and 812 ± 39 versus 757 ± 49 g for the control group, respectively). This represents a 6.6–9.6% increase in BW for all of the YCW birds over the control birds. The WG of broilers in T2, T3, T4 and T5 treatments was higher (*P* < 0.05) than the WG of broilers in the control (T1) group (776 ± 53, 786 ± 50, 765 ± 38, 768 ± 38 and 713 ± 49 g, respectively). The YCW treatments improved WG with 7.3–10.2% more gain than control birds. There were no differences (*P* > 0.05) in P‐F:G, C‐F:G, FCR or PI at day 21 of age. There was no significant difference in mortality between treatments during the starter phase. Only three birds died in T4, and one bird in each of T1 and T3 in this phase.

**Table 2:** Influence of YCW 1 and 2 and two supplementation levels on broiler performance during the 21‐day starter phase (mean ± standard deviation)

Dependent variables*	Treatment groups				
	T1	T2	T3	T4	T5
	Control	YCW‐1		YCW‐2	
		250 ppm	500 ppm	250 ppm	500 ppm
BW (g)	757 ± 49^b^	819 ± 54^a^	830 ± 51^a^	807 ± 38^a^	812 ± 39^a^
WG (g)	713 ± 49^b^	776 ± 53^a^	786 ± 50^a^	765 ± 38^a^	768 ± 38^a^
F:G	1.37 ± 0.17	1.37 ± 0.02	1.35 ± 0.03	1.35 ± 0.02	1.36 ± 0.02
FCR	1.29 ± 0.15	1.30 ± 0.02	1.28 ± 0.02	1.28 ± 0.02	1.29 ± 0.02
PI	266 ± 40	285 ± 21	292 ± 23	281 ± 17	284 ± 16
Mort (%)	0.52	0.0	0.52	1.56	0.0

^a‐b^Means ± Standard deviation within a row lacking a common superscript differ (*P* < 0.05).

* BW, body weight; WG, weight gain; F:G, feed to gain ratio; FCR, feed to weight; PI, productivity index (livability (%) x BW (kg)/Age (d)/FCRx100); Mort, percent mortality.

At the end of the 21‐ to 35‐day grower phase (Table 3), improvements on BW and WG were similar to the starter phase, and additional improvement was noticed in other performance variables such as F:G, C‐F:G, FCR and PI. The supplementation of broiler diets with either of the YCW products significantly improved BW. The increase in average BW for the grower phase was 5.4–7.8% more in YCW treatments than with the control. The average BW of the birds in T1 was 2140 ± 81 g, T2 was 2284 ± 102 g, T3 was 2307 ± 95 g, T4 was 2296 ± 85 g and T5 was 2256 ± 88 g. The WG was significantly higher in T2, T3, T4 and T5 than control (1465 ± 63, 1478 ± 67, 1489 ± 60, 1445 ± 68 and 1384 ± 55 g, respectively). Birds in T4 demonstrated significant improvement in F:G (1.55 ± 0.02) and FCR (1.45 ± 0.01), and C‐F:G (1.48 ± 0.01) was numerically lower (*P* = 0.057) than all other treatments. Productivity index (PI) was improved (*P* < 0.05) in all YCW treatments (409–422 ± 21) compared to control (389 ± 25). No significant difference was observed in mortality in the grower phase. One bird died in each of T1, T3 and T4, and two birds died in T5.

**Table 3:** Influence of YCW 1 and 2 and two supplementation strategies on broiler performance during the 35‐day grower phase (mean ± standard deviation)

Dependent variables^b^	Treatment groups				
	T1	T2	T3	T4	T5
	Control	YCW‐1		YCW‐2	
		250 ppm	250 ppm	250 ppm	250 ppm
BW (g) 1–35 days	2140 ± 81^b^	2284 ± 102^a^	2307 ± 95^a^	2296 ± 85^a^	2256 ± 88^a^
WG (g) 21–35 days	1384 ± 55^b^	1465 ± 63^a^	1478 ± 67^a^	1489 ± 60^a^	1445 ± 68^a^
F:G 21–35 days	1.59 ± 0.05^a^	1.59 ± 0.03^a^	1.59 ± 0.03^a^	1.55 ± 0.02^b^	1.60 ± 0.03^a^
C‐F:G^a^ 1–35 days	1.52 ± 0.06^a^	1.51 ± 0.02^a^	1.50 ± 0.02^a^	1.48 ± 0.01^b^	1.52 ± 0.02^a^
FCR 1–35	1.49 ± 0.06^a^	1.49 ± 0.02^a^	1.48 ± 0.02^a^	1.45 ± 0.01^b^	1.49 ± 0.02^a^
PI 1–35 days	389 ± 25^b^	419 ± 21^a^	422 ± 23^a^	422 ± 21^a^	409 ± 20^a^
Mort (%) 21–35 days	0.52	0.0	0.52	0.53	1.0
C‐Mort (%) 1–35 days	1.0	0.0	1.0	2.1	1.0

^a‐b^Means ± Standard deviation within a row lacking a common superscript differ (P < 0.05).

a *P*‐value = 0.057.

b BW, body weight; WG, weight gain; F:G, feed to gain ratio; C‐F:G, cumulative feed to gain; FCR, feed to weight; PI, productivity index (livability (%) x BW (kg)/Age (d)/FCRx100); Mort, percent mortality; C‐Mort, cumulative percent mortality.

At the end of the 35‐ to 42‐day finisher phase (Table 4), birds in any of the YCW treatments had significantly higher BW and PI than control birds. The average BW in control birds was 2875 ± 75 g, lower (*P* < 0.05) than the average BW of T2, T3, T4 and T5 birds (3032 ± 110, 3073 ± 126, 3045 ± 91 and 3013 ± 103 g, respectively). The PI of the control group (415 ± 17) was lower (P < 0.05) than the PI values of T2, T3, T4 and T5 (440 ± 20, 447 ± 24, 442 ± 15 and 433 ± 19, respectively). As observed in the early phases of this study, there was again no significant difference in finisher phase mortality, or cumulative mortality for the entire study. The total mortality (1 to 42 days) for each treatment was two birds in T1 (1.04%), one bird in T2 (0.52%), two birds in T3 (1.04%), four birds in T4 (2.08%) and three birds in T5 (1.56%).

**Table 4:** Influence of YCW 1 and 2 and two supplementation strategies on broiler performance during the 42‐day finisher phase (mean ± standard deviation)

Dependent variables^a^	Treatment groups				
	T1	T2	T3	T4	T5
	Control	YCW‐1		YCW‐2	
		250 ppm	125 ppm	250 ppm	125 ppm
BW (g) 1–42 days	2875 ± 75^b^	3032 ± 110^a^	3073 ± 126^a^	3045 ± 91^a^	3013 ± 103^a^
WG (g) 35–42 days	733 ± 39	745 ± 34	763 ± 45	747 ± 39	754 ± 28
F:G 35–42 days	1.98 ± 0.11	2.00 ± 0.08	1.97 ± 0.05	1.99 ± 0.07	1.97 ± 0.06
C‐F:G 1–42 days	1.63 ± 0.05	1.63 ± 0.02	1.62 ± 0.02	1.61 ± 0.02	1.63 ± 0.02
FCR 1–42	1.61 ± 0.05	1.61 ± 0.02	1.60 ± 0.02	1.58 ± 0.02	1.61 ± 0.02
PI 1–42 days	415 ± 17^b^	440 ± 20^a^	447 ± 24^a^	442 ± 15^a^	433 ± 19^a^
Mort (%) 35–42 days	0.0	0.5%	0.0	0.0	0.52%
C‐Mort (%) 1–42 days	1.0%	0.5%	1.0%	2.1%	1.6%

^a‐b^Means ± Standard deviation within a row lacking a common superscript differ (*P* < 0.05).

a BW, body weight; WG, weight gain; F:G, feed to gain ratio; C‐F:G, cumulative feed to gain; FCR, feed to weight; PI, productivity index (livability (%) x BW (kg)/Age (d)/FCRx100); Mort, percent mortality; C‐Mort, cumulative percent mortality.

## Discussion

This study was conducted to assess the influence of supplementation with two YCW products derived from *Saccharomyces cerevisiae*. The supplementation of YCW was conducted using two different inclusion regimens namely: supplementing the diets with YCW at a consistent level, 250 ppm, during three rearing phases, or adding YCW to the diet at three levels starting with 500 ppm in the starter phase, 250 ppm in the grower phase and 125 ppm in the finisher phase. The birds were placed in floor pens on well‐used pine‐shaving litter to mimic industry‐type rearing conditions.

The results of this study demonstrated improved performance in broilers fed YCW prebiotics using well‐used litter as a ‘natural’ challenge similar to commercially produced broilers. It was reported in previous studies that dietary concentration of supplemental YCW can have a quadratic effect on starter broiler BW subjected to Cp challenge (Fowler *et al*. 2015b), and it was calculated that the optimum YCW inclusion rate was 295 ppm.

In a different study, Fowler *et al*. (2015a) reported no significant difference in broiler performance between control corn/soybean diet and YCW treatments at 250 ppm during a 3‐week starter phase when birds were raised on fresh pine shavings. However, this study reported a significantly higher WG in birds fed YCW at 250 ppm than control birds during the grower phase. Additionally, M'Sadeq *et al*. (2015) added YCW to the diets at 800, 400 and 200 ppm during the starter, grower and finisher phases, respectively. YCW improved BW on days 24 and 35 when birds were challenged with attenuated *Eimeria spp* oocysts on day 9 and *Clostridium perfringens* on days 14 and 15. Their study concluded YCW extract, zinc bacitracin and salinomycin were effective in preventing the performance decline from necrotic enteritis and that YCW extract has promise as a tool for controlling necrotic enteritis.

The enhanced performance could be due to the overall improvement in gut health that was reported in several studies when YCW or its components were added to broiler feed such as improving intestinal integrity, gut microbiota and metabolic pathways (Shao *et al*. 2013; M'Sadeq *et al*. 2015; Tian *et al*. 2016; Hashim *et al*. 2018). Also, supplementation of YCW in broiler feed was reported to improve feed utilization especially when birds were under the stress of ingredients variations (Fowler *et al*. 2015a). These previously published studies and the current study indicate that YCW can improve broiler performance when birds are subjected to gastrointestinal stress. Total mortality at the end of the experiment did not exceed 2.1% which is very good by industry standards. No significant difference was observed in mortality between treatments.

## Conclusions and applications

The YCW products improved broiler performance when fed at a constant 250 ppm or phase fed at 500 (starter), 250 (grower) and 125 (finisher) ppm. The findings of the current study suggest that YCW prebiotic can improve broiler performance under commercial‐type rearing, using reused litter as a natural microbial challenge.

## Source of funding

This work was funded by a Phileo‐Lesaffre Animal Care grant.

## Conflict of interest

The authors declare no conflict of interest related to this report.

## Ethical statement

The authors confirm that the ethical policies of the journal, as noted on the journal's author guidelines page, have been adhered to and the appropriate ethical review committee approval has been received. The study was conducted at the Poultry Science Research Center and was approved by the Texas A&M Institutional Animal Care and Use Committee (IACUC 2014‐0030).
